# Tunable sound transmission at an impedance-mismatched fluidic interface assisted by a composite waveguide

**DOI:** 10.1038/srep34688

**Published:** 2016-10-04

**Authors:** Hui Zhang, Zhi Wei, Li Fan, Jianmin Qu, Shu-yi Zhang

**Affiliations:** 1 Key Laboratory of Modern Acoustics, MOE, School of Physics, Nanjing University, Nanjing 210093, China; 2 Department of Mechanical Engineering, Tufts University, Medford, Massachusetts 02155, USA

## Abstract

We report a composite waveguide fabricated by attaching a coupling aperture to a waveguide. The acoustic impedance of the composite waveguide can be regulated by merely controlling its coupling vibrations, depending on its structure size. By changing the size to adjust the acoustic impedance of the composite waveguide at an impedance-mismatched fluidic interface, tunable sound transmission at the desired frequencies is achieved. The reported composite waveguide provides a new method for sound regulation at a mismatched fluidic interface and has extensive frequency hopping and frequency agility applications in air-water sound communication.

Optical gratings have attracted substantial attention for their potential in sub-wavelength imaging and extraordinary transmission^1^,^2^. Inspired by these gratings, many researchers have investigated the properties of acoustic gratings with periodic arrays of sub-wavelength slits^3^,^4^,^5^. Because of the interactions among the slits in the array, the sound transmission through a sub-wavelength slit array provides diversified potential applications in sound regulation and sound imaging. Recently, the sound transmission through sub-wavelength slits or holes in a uniform fluid has attracted considerable attention. In earlier studies, Wilson and Soroka found that the sound transmission enhancement could be increased to 100 times relative to sound that was directly incident on the aperture area by using a large ratio of aperture length to aperture cross-sectional size^6^. Subsequently, Lu and Christensen revealed that the coupling effect between the diffractive wave and the waveguide mode plays an important role in this extraordinary sound transmission^3^,^4^.

Moreover, Hou found that the extraordinary sound transmission induced by Fabry–Pérot resonance could be tuned using the diffraction evanescent waves in the periodic array of subwavelength slits^7^. The evanescent field components of a subwavelength object can be efficiently transmitted through the slit array via coupling with Fabry–Pérot resonances inside the holey plate, thereby achieving acoustic imaging at the deep-subwavelength scale^8^,^9^. The acoustic magnifying hyperlens and sound suppression at Fabry–Pérot resonances can be obtained by changing the slit cross-sectional shape in the array^5^,^10^,^11^. However, the sound transmission at low frequencies stemming from Fabry–Pérot resonances always requires a large structure size. In applications, the use of a spiral channel in the tabular structure is suitable for minimizing the structure thickness, and the sound transmission and wavefront can be manipulated by controlling the phases from different spiral channels^12^,^13^.

Here, we show how the techniques can be applied to the challenge of sound transmission at an impedance-mismatched interfaces. The traditional impedance-matching method for the mismatched interface can be implemented by using a matching layer with a specific thickness and a specific acoustic impedance^14^. However, in many applications, finding appropriate matching materials is difficult. To overcome this issue, many researchers have designed structures to adjust the acoustic impedance and realize impedance matching at mismatched fluidic interfaces. Norris and Luo found that a porous solid with an array of apertures could produce a hardening effect^15^. He and Li fabricated a stiff plate combined with a periodic structure that achieved sound transmission aided by excitation of the Lamb modes^16^,^17^.

Fleury designed an acoustic metamaterial to realize impedance matching at different acoustic sides by adjusting the local acoustic property of the matching layer^18^. Similarly, Jing constructed a complementary layer with a negative acoustic property to cancel out an aberrating layer and thereby increase the sound transmission^19^. In addition, Aguanno^20^ fabricated a metamaterial comprising sub-wavelength apertures for impedance matching by controlling the filled ratio of the perforated plate or adjusting the incident angle of the sound beam. Impedance matching at mismatched fluidic interfaces can also be realized by using an aperture with a tapered profile; however, this approach inevitably induces energy attenuation and weakens the sound transmission because higher modes are prevented from traveling to standing waves^21^.

In this work, we propose a new strategy for enhancing sound transmission at an impedance-mismatched fluidic interface. To this end, a composite waveguide coupling with a closed aperture was investigated.

## Result

**Figure 1:**
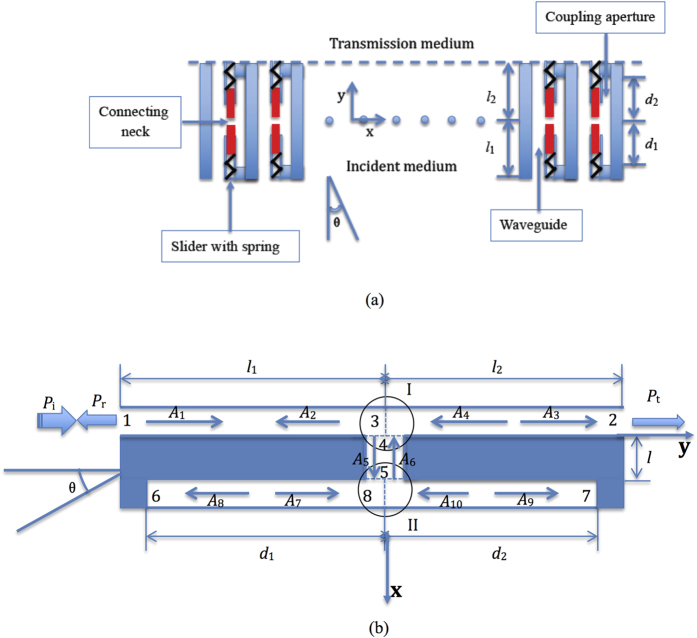
Schematic diagram of the array with the composite. (**a**) The location of the connection neck can be adjusted by two sliders attached to springs, the waveguide length *l* is divided into *l*_*1*_ and *l*_2_ by the connecting neck, and the coupling aperture length *d* is divided into *d*_*1*_ and *d*_2_ by the connecting neck. (**b**) A_1_-A_9_ represent the amplitudes of the plane waves in the composite waveguide, and circles I and II show the intersections of the connecting neck with the waveguide and the coupling aperture.

We designed a slit array in which each slit is a composite waveguide that enabled tunable sound transmission at an air-water interface. In this composite waveguide, the length in the *y* direction of the coupling aperture, as shown in Fig. 1, is comparable to the wavelength, i.e., many standing wave resonances exist in the coupling aperture. Thus, the composite waveguide produces many resonant states that are useful for regulating the acoustic impedance by controlling the coupling resonances between the coupling aperture and the waveguide.

Figure 1 schematically shows how tunable sound transmission can be achieved at an impedance-mismatched fluidic interface. In this case, each composite waveguide is directly connected to two different fluidic media at the incident and transmission fields. Meanwhile, a closed coupling aperture is designed to couple the waveguide via the connecting neck. In the coupling aperture, two sliders attached to a spring are used to adjust the connecting neck location by applying an electro-magnetic force.

Because the length of the composite waveguide is similar to the wavelength, we can describe it using a general expression of acoustic pressure and volume velocity^22^


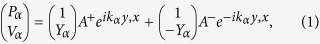


where the subscripts α = *t*, *n,* and *c* denote the waveguide, the coupling aperture, and the connecting neck in the composite waveguide, respectively. *A* + and *A***−** represent the amplitudes of the plane wave in the positive and negative directions, respectively. 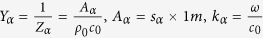 and *s*_*α*_ denote the acoustic admittance, normalized cross-sectional area, wave vector and width of the composite waveguide, respectively; *ω* = 2*πf*. As shown in Fig. 1(b), the waveguide and the coupling aperture are divided into two parts by the connecting neck. Thus, we can describe the amplitudes of the plane waves in two parts of the waveguide by (*A*_1_ and *A*_2_) and (*A*_3_ and *A*_4_), and we can describe the amplitudes of the plane waves in two parts of the coupling aperture in terms of (*A*_7_ and *A*_8_) and (*A*_9_ and *A*_10_). The amplitudes of the plane wave in the connecting neck are indicated by *A*_5_ and *A*_6_.

The acoustic impedance in a fluid medium is defined by the ratio of the sound pressure to the volume velocity; thus, the acoustic impedance of the coupling aperture can be obtained as


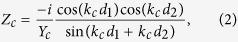


where 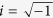, and *d*_1_ and *d*_2_ denote the lengths of two parts of the coupling aperture divided by the connecting neck. According to the impedance transfer equation in the waveguide, the acoustic impedance at the intersection of the connecting neck and the waveguide is given by


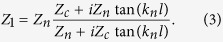


Generally, the connecting neck length *l* is usually designed to be less than one wavelength to minimize the structure size. Correspondingly, Z_1_ can be approximately described by the acoustic impedance Z_*c*_.

Considering the effects of the coupling aperture, the sound pressure transmission coefficient *T*_*P*_ through the composite waveguide can be written as


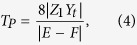


where 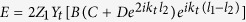; 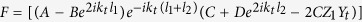, 
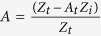
, 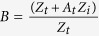; 
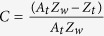
, 
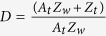
; 
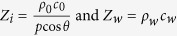
, where *p* and *θ* are the grating periodicity and the incident sound beam angle, respectively; *ρ*_0_ (*c*_0_) and *ρ*_*w*_ (*c*_*w*_) are the densities (sound speeds) on the incident side and the transmission side, respectively; and *l*_1_ and *l*_2_ are the lengths of the two parts of the waveguide divided by the connecting neck.

For the composite waveguide array, the energy transmission coefficient can be obtained as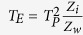. Theoretically, if the total sound energy is transmitted through the mismatched fluidic interface, 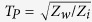 should be satisfied. Thus, Eq. (4) can be written as


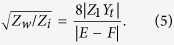


In practical applications, *Z*_*i*_ and *Z*_*w*_ are known in advance. To satisfy Eq. (5), the values of *Z*_1_, *E* and *F* must be adjusted. The adjustment of these parameters can be realized by simply changing the location of the connecting neck. More detailed investigations are described below.

The composite waveguide is assumed to have a uniform width, i.e., *s*_*α*_ = *s*_*t*_ = *s*_*n*_ = *s*_*c*_. Considering the applications of sound communication between air and water, we assume that the densities (sound speeds) on the incident side, i.e., air, and the transmission side, i.e., water, are *ρ*_0_ = 1.21 *kg*/*m*^3^ (*c*_0_ = 344 m/s) and *ρ*_*w*_ = 998 *kg*/*m*^3^ (*c*_*w*_ = 1450 m/s), respectively. Thus, the parameters are *C* ≈ 1 and *D *≈ 1 for 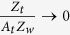. Consequently, the pressure transmission coefficient *T*_*P*_ in Eq. (4) can be simplified as


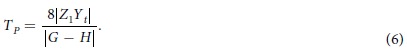


where 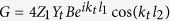,


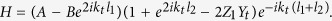


*A* and *B* are known to be finite and determined by *Z*_*i*_, *Z*_*w*_ and *s*_*t*_. If the energy transmission coefficient 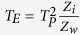 approaches unity, then *T*_*P*_ approaches infinity for 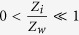. Therefore, based on Eq. (6), the pressure transmission coefficient is maximized when (*G* − *H*) → 0 and *Z*_1_ ≠ 0. To achieve this, the following relationships must be satisfied:


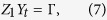



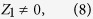


where 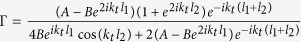.

**Figure 2:**
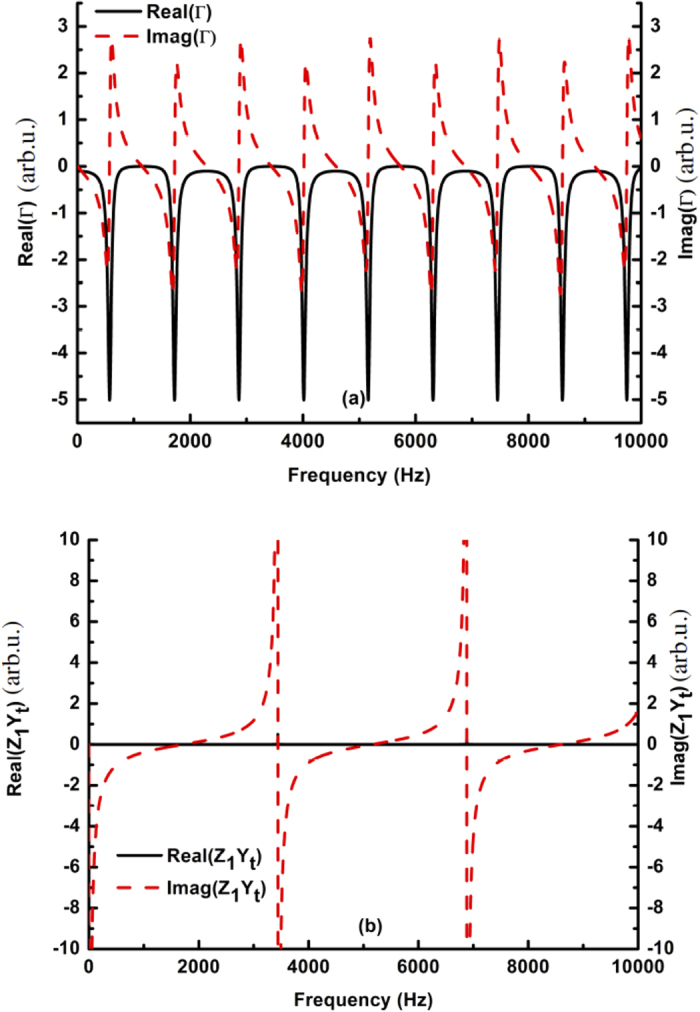
Characteristics of the acoustic impedance of the composite waveguide. In the calculations, the parameters in (**a**,**b**) were chosen to be *l*_1_ = 75 *mm*, *l*_2_ = 75 *mm*, *d*_1_ = 50 *mm*, *d*_2_ = 50 *mm* and *s*_*α*_ = 5 *mm*. (**a**) Variation of the real parts of Γ and *Z*_1_*Y*_*t*_ with the frequency. (**b**) Variation of the imaginary parts of Γ and *Z*_1_*Y*_*t*_ with the frequency.

As shown in Eqs (7) and (8), Z_1_ describes the impedance property of the coupling aperture, which has standing wave resonances of *k*_*c*_(*d*_1_ + *d*_2_) = *mπ (m* = 1, 2, 3, …). The resonant states are shown in Fig. 2 and reveal how the real and imaginary parts of Z_1_ vary with the frequency. However, coupling effects exist between the coupling aperture and the waveguide. Although *k*_*c*_(*d*_1_ + *d*_2_) = *mπ* is satisfied, the standing wave resonances may not be excited effectively when the connecting neck is simply placed at the antinode of the standing wave velocity field in the coupling aperture. This phenomenon can be ascribed to the fact that the velocity relation *A*_7_ + *A*_8_ = *A*_9_ − *A*_10_ is no longer satisfied because the connecting neck adds a constraint condition: 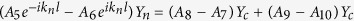. Detailed theoretical descriptions are provided in the method section.

In contrast, based on Eq. (7), the parameter Γ mainly depends on *l*_1_, *l*_2_, *A* and *B*, and *l*_1_ and *l*_2_ affect the resonant states of the waveguide. For a waveguide at a mismatched fluidic interface, the cavity resonances can be excited when *k*_*t*_(*l*_1_ + *l*_2_) = 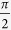 (2*n*−1) (*n* = 1, 2, 3, …) because of the large impedance mismatch between air and water. Similar to the coupling aperture, the resonant states in the waveguide may also be weakened when the connecting neck is placed at the antinode of the standing wave velocity field in the waveguide.

Clearly, *Z*_1_ and Γ are closely related to the resonant states in the coupling aperture and the waveguide. In addition, *Z*_1_ and Γ have different variation tendencies. For example, the real and imaginary parts of Γ and *Z*_1_*Y*_*t*_ exhibit different periodic variations with the frequency, as shown in Fig. 2. More interestingly, their real parts are almost zero, except in some resonance states. Furthermore, the imaginary parts exhibit monotonous variation between two adjacent resonant states. In other words, the real parts on both sides of Eq. (7) become zero when the frequencies are distant from the resonant states. When designing tunable sound transmission, the relationships *Z*_1_*Y*_*t*_ = Γ and *Z*_1_ ≠ 0 can be satisfied at the non-resonant states by simply adjusting the non-zero imaginary parts on both sides of Eq. (7). Note that the location of the connecting neck can change the coupling effects in the composite waveguide and influence imaginary parts of *Z*_1_ and Γ.

**Figure 3:**
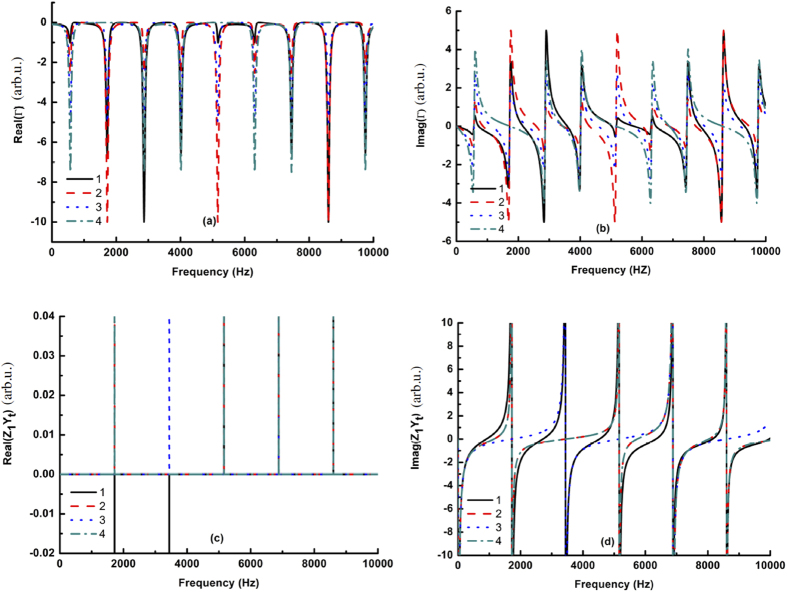
Effects of the connecting neck location on the impedance characteristics of the composite waveguide. (**a**,**b**) show the variations of the real and imaginary parts of Γ with the connecting neck location. (**c**,**d**) show the variations of the real and imaginary parts of *Z*_1_*Y*_*t*_ with the connecting neck location. In (**a**–**d**), **1** denotes *l*_1_ = 30 *mm*, *l*_2_ = 120 *mm*, *d*_1_ = 5 *mm*, *d*_2_ = 95 *mm* and *s*_*α*_ = 5 *mm*; **2** denotes *l*_1_ = 50 *mm*, *l*_2_ = 100 *mm*, *d*_1_ = 25 *mm*, *d*_2_ = 75 *mm* and *s*_*α*_ = 5 *mm*; **3** denotes *l*_1_ = 75 *mm*, *l*_2_ = 75 *mm*, *d*_1_ = 50 *mm*, *d*_2_ = 50 *mm* and *s*_*α*_ = 5 *mm*; and **4** denotes *l*_1_ = 100 *mm*, *l*_2_ = 50 *mm*, *d*_1_ = 75 *mm*, *d*_2_ = 25 *mm* and *s*_*α*_ = 5 *mm*.

To understand the characteristics of tunable sound transmission at different frequencies, we investigated the effects of the neck location on the transmission frequencies. Figure 3 shows that as the neck location changes, the imaginary part of Γ varies continuously between two adjacent resonance states. This behavior is different from that exhibited by a waveguide at an air-water interface because the coupling aperture has the same rigid boundary conditions on both end-sides, which produces similar imaginary part curves of *Z*_1_*Y*_*t*_ when the neck is symmetrically placed along the coupling aperture, as shown in curves 2 and 3 in Fig. 3(c,d). Therefore, the neck location changes *Z*_1_ and Γ, and as a result, *Z*_1_*Y*_*t*_ = Γ and *Z*_1_ ≠ 0 can be satisfied at different frequencies by varying the neck location.

In practical applications, tunable sound transmission can be accomplished by changing the neck location through the use of an electro-magnetic force to drive the two sliders attached to the spring. For example, consider a case in which the outer dimension of the composite waveguide is fixed: The aperture width is *s*_*α*_ = 5 *mm*, the array period is *p* = 50 *mm*, the length of the connecting neck is *l *=* *5 *mm*, the length of the main aperture is *l*_1_ + *l*_2_ = 150 *mm*, and the length of the coupling aperture *d*_1_ + *d*_2_ = 100 *mm*. Thus, we can adjust the local dimensions, such as *l*_1_, *l*_2_, *d*_1_ and *d*_2_, by controlling the location of the connecting neck. When the neck location is shifted from the middle position to the end position of the coupling aperture, total sound transmission at the lowest frequency can be adjusted from 1230 Hz to 1315 Hz. The tunable frequency range of total sound transmission could be further increased by also controlling the length of the coupling aperture.

## Discussion

**Figure 4:**
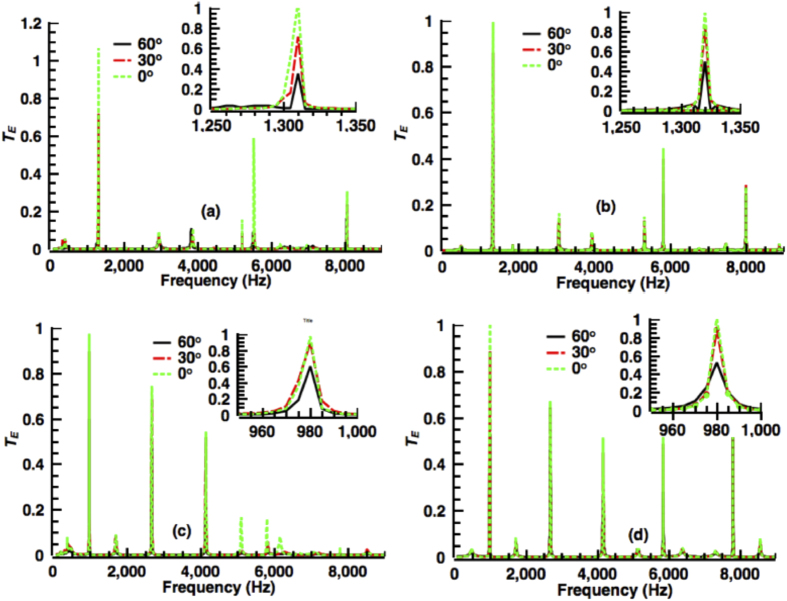
Transmission coefficients of the composite waveguide array with the sound beam incident at 0 and 30 and 60 degrees. The insets represent the variations of the energy transmission at the first peak frequency with the different incident angles. (**a**) Transmission coefficients obtained from the FEM simulation. (**b**) Transmission coefficients obtained from the theoretical model. In (**a**,**b**), the parameters were chosen as *l*_1_ = 75 *mm*, *l*_2_ = 75 *mm*, *d*_1_ = 50 *mm*, *d*_2_ = 50 *mm* and *s*_*α*_ = 5 *mm* (**c**) Transmission coefficients obtained from the FEM simulation. (**d**) Transmission coefficients obtained from the theoretical model. In (**c**,**d**), the parameters were chosen as *l*_1_ = 50 *mm*, *l*_2_ = 100 *mm*, *d*_1_ = 25 *mm*, *d*_2_ = 75 *mm* and *s*_*α*_ = 5 *mm*.

To further confirm the theoretical analysis described above, we used the finite element method (FEM) to construct an array with 20 composite waveguide elements for potential applications in air-water communication^23^ or sound transmission enhancement in medical ultrasound and nondestructive evaluations^24^,^25^. For FEM, a 2-D model was constructed from an aluminum plate with a coupling aperture array. The incident medium and transmission medium were chosen to be air and water, respectively. The FEM results were then compared with those obtained using Eq. 4. Figure 4 shows that the FEM simulation results are in good agreement with our theoretical analysis when the frequency was less than 5000 Hz. The low-frequency range is suitable for sound information transfer in air-water communication. In the high-frequency range, the FEM results deviate from the theoretical results. This deviation is attributable to two main reasons: (1) The higher-order diffraction modes of the array cannot be ignored in the high-frequency range but were not considered in the theoretical analysis. (2) The finite dimension of the array with 20 elements used in the FEM model might lead to boundary effects that disturb the acoustic field.

Moreover, the theoretical analysis requires 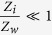, and this condition is difficult to satisfy as the incident angle increases. In general, *T*_*E*_ decreases as the incident angle increases. Here, we investigate the sound transmission at the first frequency with the maximum *T*_*E*_ to reveal the relationship between *T*_*E*_ and *θ*. The highest energy transmission occurs at the first peak, which is the most typically used frequency in air-water communication applications. The insets in Fig. 4 represent the variations of the energy transmission at the first peak frequency as the incident angle changes. Figure 4 shows that *T*_*E*_ is approximately 0.5 when the incident angle is 60 degrees, i.e., *T*_*E*_ is roughly proportional to cos *θ*. In addition, other energy transmission peaks show similar relationships with the incident angle. Interestingly, the frequencies of the transmission peaks do not change with the incident angle, unlike the impedance matching achieved using a perforated metamaterial. Because of this unique property, the composite waveguide can be used over a wide range of incident angles.

In conclusion, a composite waveguide was designed to realize tunable sound transmission. Our theoretical analysis demonstrated that the adjustable connecting neck can be used to regulate sound transmission at different frequencies by changing the coupling resonant states between the coupling aperture and waveguide. Meanwhile, the sound transmission peak is largely retained over al large range of incident angles, facilitating considerable energy transmission and facilitating sound control over a large range of angles. Additionally, the waveguide and coupling aperture can be fabricated with zigzag shapes to create resonant states with lower frequencies, which would be useful for air-water communication applications.

## Methods

### Theory

The composite waveguide, which is shown in Fig. 1(b), can be described by the nonlocal theory reported in ref. 22. Because of the continuity of the sound pressure and volume velocity in cross-section 1, which is on the incident side of the waveguide, we obtain the following relationships:


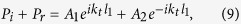



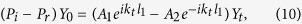


where 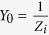.

In junction I, which is shown in circle I of Fig. 1(b), at the connection position of the waveguide and the neck, the sound pressure and volume velocity should satisfy


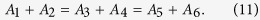



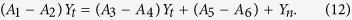


For cross-section 2 of the waveguide, which is on the transmission side, the sound pressure and volume velocity must be continuous and satisfy


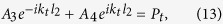



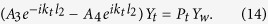


where 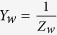.

For simplicity, we assume that 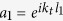 and 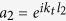. Therefore, Eqs (13) and (14) can be rewritten as 
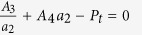
 and 
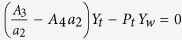
, respectively. Thus, we can obtain


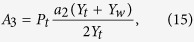



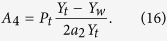


According to Eqs (9) and (10), the following relationship can be obtained:


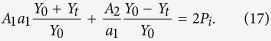


From Eq. (11), *A*_1_ = *A*_3_ + *A*_4_ − *A*_2_; substituting this equation into Eq. (17) yields *A*_2_:


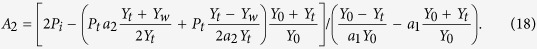


According to Eqs (15), (16) and (18) and *A*_1_ = *A*_3_ + *A*_4_ − *A*_2_, the constant *A*_1_ can be written as


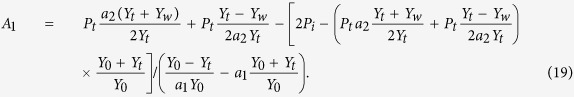


Next, we divide Eq. (12) by Eq. (11) to obtain the following relationship:


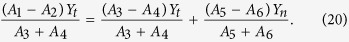


In Eq. (20), we write 
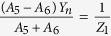
, where *Z*_1_ represents the acoustic impedance at cross-section 4 of the connecting neck linked to the waveguide, which can be obtained by investigating sound propagation in the coupling aperture and the connecting neck. Two rigid boundary conditions exist in cross-sections 6 and 7 of the coupling aperture, and the volume velocity on the boundaries should be zero. Thus, the following relationships can be obtained:


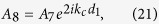



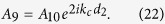


However, the sound pressures on both sides of cross-section 8 in the coupling aperture are equal, resulting in the following:


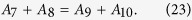


Substituting Eqs (21) and (22) into Eq. (23) gives the following relationship:


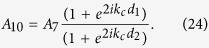


Similar to junction I, the sound pressure and volume velocity in junction II, as shown in circle II of Fig. 1(b), satisfy the following relationships:


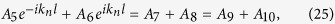



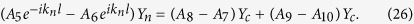


Based on Eqs (25) and (26), the acoustic impedance in cross-section 5 of the connecting neck is found to be


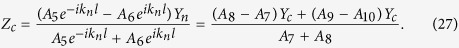


Substituting Eqs (21), (22) and (24) into Eq. (27) gives


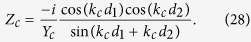


Furthermore, according to the impedance transfer relationship, the acoustic impedance Z_1_ at cross-section 4 of the connecting neck can be expressed as


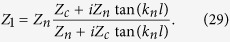


Finally, substituting Eqs (15), (16), (18), (19) and (29) into Eq. (20) produces the transmission coefficient of the sound pressure in the composite waveguide:


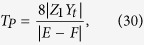


where 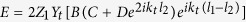 ; 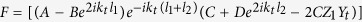 ; 
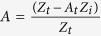
, 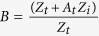, 
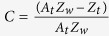
, 
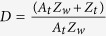
. Correspondingly, the energy transmission coefficient can be obtained: 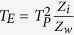.

### FEM simulations

We constructed the FEM model using COMSOL MULTIPHYSICS with a 20-element array. In our analyses, the mass density, Young’s modulus and Poisson’s ratio of the aluminum plate were 2700 kg/m^3^, 6.85 × 10^10^ Pa and 0.34, respectively. The relevant values for air are as follows: *ρ*_0_ = 1.21 kg/m^3^, ambient pressure = 1 atm, and sound speed *c*_0_ = 343 m/s. The relevant values for water are as follows: *ρ*_w_ = 998 kg/m^3^ and sound speed *c*_w_ = 1450 m/s.

In the simulation, the following parameters were used: grating periodicity *p* = 50 *mm*, *ρ*_0_*c*_0_ = 0.42 MRayls and *ρ*_*w*_*c*_*w*_ = 1447 MRayls. The sound pressure of the incident beam was fixed at 1 Pa. The pressure transmission coefficient *T*_*P*_ can be obtained as the ratio of the sound pressures of the transmission field and the incident field, and the maximum sound pressure in the transmission field is chosen because the transmitted sound pressure decreases with the radiated distance in the free transmission field. The energy transmission coefficient *T*_*E*_ can be obtained using the relation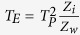. The results obtained using our theoretical model and FEM were normalizing, and the energy transmission coefficient was obtained by dividing the transmission spectrum by the maximum energy transmission coefficient.

## Additional Information

**How to cite this article**: Zhang, H. *et al.* Tunable sound transmission at an impedance-mismatched fluidic interface assisted by a composite waveguide. *Sci. Rep.* **6**, 34688; doi: 10.1038/srep34688 (2016).
